# Characterization of the χψ subcomplex of *Pseudomonas aeruginosa *DNA polymerase III

**DOI:** 10.1186/1471-2199-12-43

**Published:** 2011-09-28

**Authors:** Sirine El Houry Mignan, Gregor Witte, Natalie Naue, Ute Curth

**Affiliations:** 1 Institute for Biophysical Chemistry, Hannover Medical School, Carl-Neuberg-Str. 1, 30625 Hannover, Germany; 2 Department of Biochemistry, Gene Center of the Ludwig-Maximilians-University Munich, Feodor-Lynen-Str. 25, D-81377 Munich, Germany; 3 Center for Integrated Protein Sciences (CIPSM), Gene Center of the Ludwig-Maximilians-University Munich, Feodor-Lynen-Str. 25, D-81377 Munich, Germany; 4 Munich Center for Advanced Photonics (MAP), Gene Center of the Ludwig-Maximilians-University Munich, Feodor-Lynen-Str. 25, D-81377 Munich, Germany

## Abstract

**Background:**

DNA polymerase III, the main enzyme responsible for bacterial DNA replication, is composed of three sub-assemblies: the polymerase core, the β-sliding clamp, and the clamp loader. During replication, single-stranded DNA-binding protein (SSB) coats and protects single-stranded DNA (ssDNA) and also interacts with the χψ heterodimer, a sub-complex of the clamp loader. Whereas the χ subunits of *Escherichia coli *and *Pseudomonas aeruginosa *are about 40% homologous, *P. aeruginosa *ψ is twice as large as its *E. coli *counterpart, and contains additional sequences. It was shown that *P. aeruginosa *χψ together with SSB increases the activity of its cognate clamp loader 25-fold at low salt. The *E. coli *clamp loader, however, is insensitive to the addition of its cognate χψ under similar conditions. In order to find out distinguishing properties within *P. aeruginosa *χψ which account for this higher stimulatory effect, we characterized *P. aeruginosa *χψ by a detailed structural and functional comparison with its *E. coli *counterpart.

**Results:**

Using small-angle X-ray scattering, analytical ultracentrifugation, and homology-based modeling, we found the N-terminus of *P. aeruginosa *ψ to be unstructured. Under high salt conditions, the affinity of the χψ complexes from both organisms to their cognate SSB was similar. Under low salt conditions, *P. aeruginosa *χψ, contrary to *E. coli *χψ, binds to ssDNA via the N-terminus of ψ. Whereas it is also able to bind to double-stranded DNA, the affinity is somewhat reduced.

**Conclusions:**

The binding to DNA, otherwise never reported for any other ψ protein, enhances the affinity of *P. aeruginosa *χψ towards the SSB/ssDNA complex and very likely contributes to the higher stimulatory effect of *P. aeruginosa *χψ on the clamp loader. We also observed DNA-binding activity for *P. putida *χψ, making this activity most probably a characteristic of the ψ proteins from the Pseudomonadaceae.

## Background

*Pseudomonas aeruginosa *is a ubiquitous Gram-negative bacterium that causes high rates of hospital-acquired infections, especially in immunocompromised patients [1] as well as in patients with cystic fibrosis [2]. *P. aeruginosa *infections are often difficult to treat because the pathogen is capable of very rapidly acquiring a multitude of resistance mechanisms [3], making multidrug resistant *P. aeruginosa *strains increasingly common and raising the need for new antipseudomonal drugs. The replication machinery of *P. aeruginosa *presents a good target for the development of new antimicrobial agents, since DNA replication is essential for the survival of the pathogen.

DNA polymerase III holoenzyme is the main enzyme involved in the replication of the bacterial genome. In *Escherichia coli*, this multiprotein complex is composed of ten distinct subunits that are arranged into three functional subassemblies [4]. The core subassembly contains the DNA polymerase and the proofreading exonuclease activities [5]. The β-sliding clamp encircles the DNA duplex while binding to the core and thereby tethering it to the DNA template, ensuring high processivity and speed of the holoenzyme [6]. The clamp loader complex, composed of subunits τ/γ, δ, δ', χ and ψ, uses ATP hydrolysis to load the clamp onto DNA [7].

The τ/γ, δ and δ' subunits together form the "minimal" clamp loader, (τ/γ)_3_δδ', which is sufficient for loading the clamp. The χ and ψ subunits are not essential for clamp loading [8]. However, χ is the only direct link between DNA polymerase III and SSB, the single-stranded DNA-binding protein [9,10] which coats single-stranded DNA (ssDNA) at the lagging strand to protect it from degradation and to prevent hairpin formation. The SSB/χ interaction is necessary for the primase-to-polymerase switch, which involves a competition between DnaG primase and χ in binding to SSB [11]. The ψ subunit bridges χ to the (τ/γ)_3_δδ' complex and has been shown to play an important role in stabilizing the clamp loader [12] and increasing its affinity for the β-clamp [13]. In *E. coli*, χ and ψ form a tight 1:1 complex, the crystal structure of which has been solved [14]. The residues at the interface between χ and ψ are highly conserved across several bacterial species [14], and the same holds true for a hydrophobic surface pocket of χ surrounded by basic residues which was shown to interact with the amphipathic C-terminus of SSB [15]. The disordered N-terminal part of ψ is also well-conserved, and is required for the binding of χψ to the minimal clamp loader [16]. However, in several bacterial genomes containing χ sequences in which the ψ-binding site appears to be conserved, ψ sequences cannot be found, suggesting that ψ has greatly diverged in these species [14].

Sequence comparisons between *E. coli *and *P. aeruginosa *identified the existence of all genes encoding the subunits of DNA polymerase III except for θ, a non-essential subunit of the core, and ψ [17]. The ψ subunit could only be identified as a 32 kDa protein which copurified with the other DNA polymerase III subunits from *P. aeruginosa *cell lysate [18]. The start codon of the gene encoding this subunit was found to be incorrectly annotated in the *P. aeruginosa *PAO1 genome, the actual start codon being a non-canonical UUG present 135 nucleotides upstream from the originally annotated one. Examination of the ψ amino acid sequence showed that it is divergent from and nearly twice the length of *E. coli *ψ. At the functional level, the *P. aeruginosa *χψ complex appears to play a more significant role than its *E. coli *counterpart. Jarvis et al. [18] observed that, under low salt conditions and at subsaturating levels of the τ_3_δδ' minimal clamp loader, the addition of *P. aeruginosa *χψ and SSB has a synergistic effect, increasing the activity of *P. aeruginosa *τ_3_δδ' 25-fold. In contrast, under similar conditions, *E. coli *τ_3_δδ' was insensitive to the addition of *E. coli *χψ [17]. At high salt concentrations, however, both χψ and SSB are required for efficient DNA synthesis, in the *P. aeruginosa *as well as in the *E. coli *systems [18].

This report describes the biophysical characterization of the χψ complex of *P. aeruginosa*, revealing properties which distinguish it from its *E. coli *counterpart and possibly account for its higher stimulatory effect. Using analytical ultracentrifugation, we show that *P. aeruginosa *ψ, but not the *E. coli *ψ subunit, binds to ssDNA under low salt conditions, even when the ssDNA is coated with SSB. This binding results in an increased affinity of *P. aeruginosa *χψ toward the SSB/ssDNA complex when compared to the *E. coli *system. In addition, we found that *P. putida *χψ also binds to ssDNA, suggesting that DNA-binding is a property of ψ proteins from the Pseudomonadaceae.

## Results and Discussion

### Biophysical characterization of *Pae*χψ

To determine the composition of *P. aeruginosa *χψ (*Pae*χψ), the complex was subjected to sedimentation equilibrium experiments in the analytical ultracentrifuge under high salt conditions. The concentration gradients obtained at three different rotor speeds (see Methods section) and two protein concentrations [5.8 μM (Additional file 1: **Figure S1 *A***) and 23.3 μM] could be globally fitted with a single molar mass of 46 (± 4) kg/mol. There was no indication of multiple species or aggregation. Since the molar mass of a *Pae*χψ heterodimer calculated from the amino acid composition is 46.2 kg/mol, the protein exists as a heterodimer in solution. In comparison, examination of the χψ complex of *E. coli *(*Eco*χψ) yielded a molar mass of 31 (± 4) kg/mol (Additional file 1: **Figure S1 *B***), confirming the formation of *Eco*χψ heterodimers (calculated molar mass 31.8 kg/mol) as reported previously [12].

To characterize its hydrodynamic properties, the *Pae*χψ heterodimer was examined in sedimentation velocity experiments in the analytical ultracentrifuge under high salt conditions. The data measured at three different protein concentrations were analyzed using the program package SEDFIT [19]. For *Pae*χψ, the *c*(*s*) distributions in a concentration range of 1.9 to 15.5 μM revealed a single species, sedimenting with an s_20, W _of 2.5 S, and gave no indication of significant impurities or aggregation products (Additional file 2: **Figure S2 *A***). Even at the lowest protein concentration used, no change in the *c*(*s*) distribution could be observed, showing that the χψ complex is stable under these conditions with no tendency to dissociate. In comparison, the *c*(*s*) analysis for *Eco*χψ resulted in s_20, W_= 2.5 S and the complex also showed no tendency to aggregate at higher or to dissociate at lower protein concentrations (Additional file 2: **Figure S2 *B***).

From the calculated molar masses of the χψ heterodimers and the measured sedimentation coefficients, the frictional ratios f/f_0 _and the hydrodynamic radii r_H _were calculated. For the *P. aeruginosa *complex this resulted in f/f_0_= 1.80 and r_H_= 4.3 nm, whereas the *E. coli *complex yielded f/f_0_= 1.42 and r_H_= 3.0 nm. Since the frictional ratios for hydrated spherical proteins are expected to be in the range of 1.1 to 1.2 [20], the shape of both protein complexes seems to deviate substantially from a sphere. For *Eco*χψ, this is in agreement with the results from the crystal structure [14], where it was shown that the complex is elongated and that the 26 N-terminal amino acids of ψ are disordered. As the frictional ratio of *Pae*χψ is even larger, a GlobPlot analysis [21] of the χ and ψ proteins was performed, which predicted the presence of two large disordered regions in the N-terminus of *Pae*ψ, while *Pae*χ was predicted to be mostly globular (data not shown).

To check the GlobPlot prediction for *Pae*ψ, a truncated version of this protein was constructed in which the 85 N-terminal amino acids were deleted. This construct was coexpressed with full-length *Pae*χ and both proteins could be copurified as a *Pae*χψ_(Δ1-85) _complex. Although the molar mass of this complex was reduced by 9.1 kg/mol due to the truncation, it sedimented essentially with the same sedimentation coefficient as observed for full-length *Pae*χψ (s_20, W_= 2.5 S, see additional file 2: **Figure S2 *C***). Accordingly, the frictional ratio and the hydrodynamic radius dropped to f/f_0_= 1.55 and r_H_= 3.4 nm, respectively. This result makes it very likely that the N-terminal part of *Pae*ψ is disordered. It is worth noting, however, that the frictional ratio of the truncated complex is still higher than that of *Eco*χψ.

### SAXS measurements reveal that *Pae*χψ is elongated in solution

To get structural information of *Pae*χψ in solution, and to examine the lack of structure within the N-terminal part of *Pae*ψ in particular, we performed small-angle X-ray scattering (SAXS) experiments of the full-length and the truncated protein complexes (Additional file 3). High-throughput crystallization attempts with both *Pae*χψ and *Pae*χψ_(Δ1-85) _unfortunately failed to produce suitable crystals for X-ray crystallography, thus no high resolution structure could be obtained.

**Figure 1:**
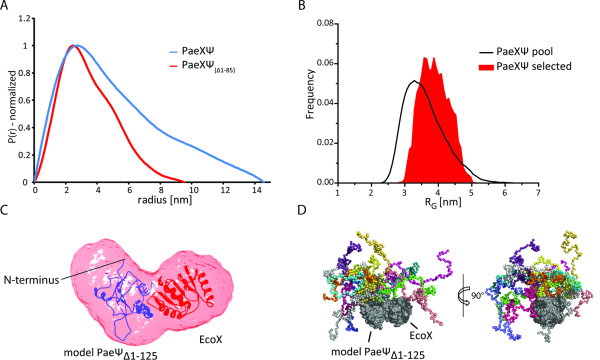
**SAXS analysis of *Pae*χψ and *Pae*χψ_(Δ1-85)_**. *(**A**) *Pair-distribution functions (normalized to equal peak height) of *Pae*χψ (*blue*) and *Pae*χψ_(Δ1-85) _(*red*) calculated with GNOM indicate that *Pae*χψ is more elongated than *Pae*χψ_(Δ1-85)_. *(**B**) *Radius of gyration distributions of pools (*black line*) and selected structures (*red area*) for the EOM analysis of full-length *Pae*χψ. *(**C**) Ab initio *shape of *Pae*χψ_(Δ1-85) _(*transparent red*) calculated with GASBOR with the modeled E*co*χP*ae*ψ-structure (see text) docked into the density. *(**D**) *Superposition of the selected structures from EOM analysis of full-length *Pae*χψ. The structures were superimposed using only the core-domains of the proteins (shown in grey for simplification of the image). The flexible N-terminal residues of Paeψ are shown in a different color for each of the selected structures. SAXS experiments were performed in 20 mM Hepes pH 7.4, 50 mM NaCl, 3% (w/v) sucrose, 1 mM NaN_3_, 1 mM DTT.

The shape of the SAXS curve for full-length *Pae*χψ directly suggests an elongated structure of the complex as it resembles a straight line in the lower s region (Additional file 3: **Figure S3 *A***) [22]. Analysis of the Guinier-plot (log I(s) vs. s^2^) for the lowest protein concentration (2 mg/ml) yielded a radius of gyration of R_G_= 4.19 nm. From extrapolation of I(s) to s= 0 and from Porod volume we calculated molar masses of 44.3 and 47.7 kg/mol, respectively. These observations are in good agreement with the theoretical mass of the *Pae*χψ heterodimer and with the results of the sedimentation equilibrium experiments. The scattering curves were used to calculate the pair-distribution function, P(r) (Figure 1A). The plot for the full-length complex is typical of elongated molecules, which are described by functions with a maximum at smaller distances and which usually show tailing at higher distances [23]. The maximum particle diameter, D_max_= 14.6 nm, indicates an elongated shape of the χψ heterodimer. The analysis of the scattering data using a Kratky-plot (Additional file 4) suggests that the full-length complex probably contains extensive unfolded regions. A test set of independent *ab initio *structures, calculated using this dataset, shows large differences among the models and thus supports the idea of a potentially highly flexible part in the complex. To test whether it is the N-terminus of *Pae*ψ which is unstructured, we performed SAXS experiments with the N-terminally shortened construct *Pae*χψ_(Δ1-85)_. This construct also forms heterodimers (M= 32 kg/mol from I(0), M= 33.6 kg/mol from Porod volume), but is obviously much more compact and does not possess large protrusions as can be deduced from the P(r)-distribution (Figure 1A) and the value of R_G_= 2.9 nm. These results are also in good agreement with the analytical ultracentrifugation experiments.

**Figure 2:**
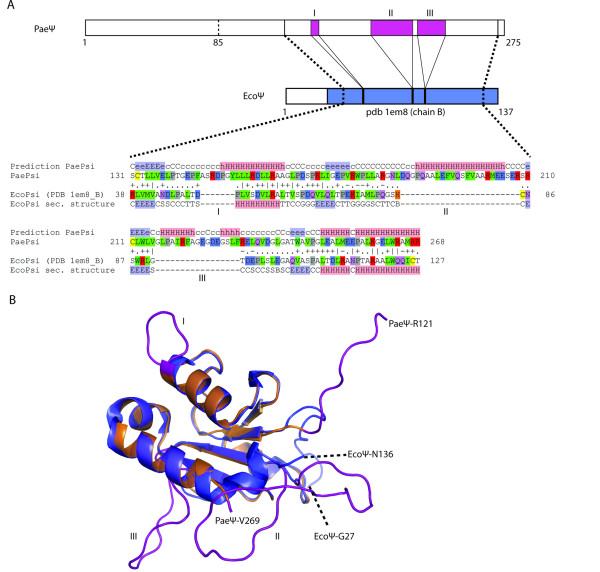
**Homology-based modeling**. (***A***) Schematic alignment of *Pae*ψ and *Eco*ψ based on HHpred results with depicted structure-homology domain (*Eco*ψ, pdb code: 1em8, chain B [14]). The corresponding HHpred alignment of the core domains is shown below and indicates a high structural homology between the C-terminal part of *Pae*ψ and *Eco*ψ. Possible loop-extensions occurring in *Pae*ψ are numbered in roman numerals. *(**B**) *Superposition of the modeled core structure of *Pae*ψ (*orange*) obtained from MODELLER on *Eco*ψ in blue (pdb code: 1em8, chain B). The insertions in the *Pae*ψ sequence do not interfere with secondary structure elements of *Eco*ψ and can be assigned to loop regions. The extended loops are colored in pink and are labeled as in (***A***).

We used the sequence of P*ae*ψ in a semi-automated homology search and modeling approach using the Bioinformatics Toolkit server [24]. The final output model obtained with the program MODELLER [25], using *Eco*ψ (pdb code: 1em8) as a template, was superimposed on the existing structure of *Eco*ψ [14]. Secondary structure prediction suggests that the *Pae*ψ N-terminus is potentially unstructured and therefore no structural relatives for this part could be identified. The additional insertions in the core of ψ, however, occur in extended or changed loop regions of *Eco*ψ (Figure 2). These insertions, namely loops II and III, are probably largely unstructured, even though it can not be ruled out that they might fold back and extend the central core β-sheet. The presence of extended loop structures would be in accordance with the higher frictional ratio of the N-terminally truncated *Pae*χψ_(Δ1-85) _compared to *Eco*χψ as measured by analytical ultracentrifugation.

Based on HHpred analysis, the core domains of *Pae*χψ and *Eco*χψ show high homologies; we therefore tested whether the *ab initio *shape calculated from *Pae*χψ_(Δ1-85) _scattering data is able to harbor the *in silico*-generated *Pae*ψ-*Eco*χ heterodimer that we modeled. As *Pae*ψ is much larger than *Eco*ψ, this model accounts for the putative presence of extended loops in *Pae*ψ as predicted from sequence alignment (Figure 2A). The shape of the final *ab initio *SAXS model of *Pae*χψ_(Δ1-85) _(Figure 1C) is clearly able to harbor both the modeled χψ-complex and the N-terminal residues 86-125 of *Pae*ψ, which are not present in our model but in the measured construct.

We further analyzed the SAXS data of the *Pae*χψ full-length complex using an alternative approach: lacking data to calculate reliable *ab initio *models due to the large and probably flexible N-terminus of *Pae*ψ, we performed an ensemble optimization method approach (EOM) [26]. This procedure creates a large pool of random configurations and a genetic algorithm is used to select ensembles being in agreement with the experimental scattering data. For this approach we used our *in silico*-generated construct of *Pae*ψ and *Eco*χ as a rigid (and folded) core and defined the absent N-terminus of *Pae*ψ as a flexible part containing 125 residues. The R_G_-distribution obtained for the models in the selected ensembles is quite broad (Figure 1B), supporting the idea of high flexibility (e.g. [27]). The chosen ensemble of structures was then superposed (Figure 1D) to visualize the different conformations of the N-terminus which all fit to the measured scattering curve. Additionally, we calculated the theoretical sedimentation coefficients of all structures in the chosen ensemble using HYDROPRO [28]. The average s-value of s_20, W _= 2.6 ± 0.1 S is in very good agreement with our experimental data.

### *Pae*χψ binds to the highly conserved C-terminus of SSB

**Figure 3:**
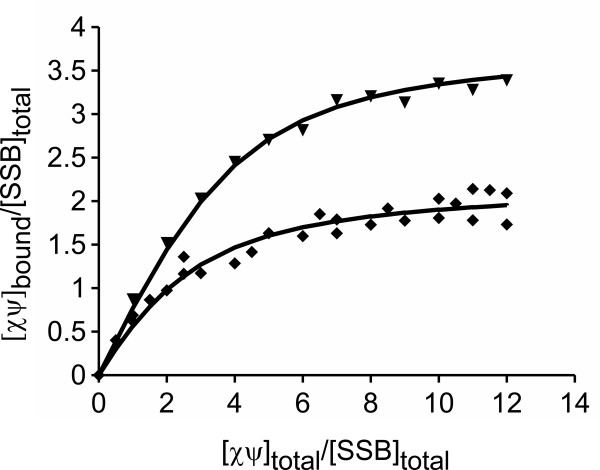
***Pae*χψ and *Eco*χψ bind with similar affinities to their cognate SSB proteins**. 6 μM SSB was sedimented in the analytical ultracentrifuge in high salt buffer, at 50000 rpm and 20°C (λ = 280 nm) in the presence of increasing concentrations of χψ. Binding isotherms for the interactions of χψ with SSB for the proteins of *P. aeruginosa *(*diamonds*) and *E. coli *(*triangles*) were obtained by analysis of the *c*(*s*) distributions (see Methods section). The solid lines represent theoretical binding isotherms calculated for a simple interaction model of *n *molecules of χψ with one SSB tetramer using the following parameters: *Pae n *= 2.2, *K *= 1.3 × 10^5 ^M^-1^; *Eco n *= 3.8, *K *= 1.8 × 10^5 ^M^-1^.

Next, we compared *Eco*χψ and *Pae*χψ at the functional level. Since *Eco*χψ is known to interact through χ with the highly conserved C-terminus of *Eco*SSB, we investigated the interaction between *Pae*χψ and P*ae*SSB. When two molecules interact, they form a complex with a larger mass which usually sediments faster than each of the components. Therefore, it is possible to analyze the interaction between χψ and SSB in sedimentation velocity experiments in the analytical ultracentrifuge, as described for the interaction of *Eco*χ and *Thermus aquaticus *SSB [29]. Because free χψ sediments slower than free SSB, *Pae*χψ was titrated to a constant concentration of *Pae*SSB in high salt buffer; for comparison, the same experiment was performed with the *E. coli *proteins. From the *c*(*s*) distributions, the concentrations of free χψ were determined, and binding isotherms were constructed using a model for independent binding of *n *χψ complexes to one SSB tetramer (Figure 3). For the binding of *Eco*χψ to *Eco*SSB, we obtained an affinity of 1.8 × 10^5 ^M^-1^, which is in accordance with previously published data on this system [30], and which is within the same range as the binding affinity of only *Eco*χ to *Eco*SSB [10], suggesting that *Eco*ψ does not play a role in the binding of *Eco*χψ to SSB.

For *Pae*χψ and *Pae*SSB, a similar binding affinity of 1.3 × 10^5 ^M^-1 ^was obtained under the same experimental conditions (Figure 3). The binding stoichiometry, however, is lower than in the case of *E. coli*. As both SSB proteins are known to form homotetramers [31,32], there exist four potential binding sites for χψ, one at the C-terminus of each monomer. Since the binding isotherm yielded a stoichiometry of *n*= 3.8 for the *E. coli *system, each C-terminus of *Eco*SSB binds one *Eco*χψ complex (Figure 3). Correspondingly, it has been shown that up to four *Eco*χ proteins can bind to *Eco*SSB [10]. In the case of *P. aeruginosa*, however, not all binding sites can be occupied (*n*= 2.2, Figure 3). This might be due to the fact that *Pae*ψ is almost twice the size of *Eco*ψ and that, as was shown above, the N-terminus of *Pae*ψ is highly elongated. Thus, it is most probably sterical hindrance that prevents more than two *Pae*χψ complexes from binding to SSB via χ.

To test whether *Pae*χψ has the same binding site on SSB as the *E. coli *complex, we used the deletion mutant *Eco*SSBQ152* [33], which is truncated of the last 26 amino acids and which has been shown to be unable to interact with *Eco*χ [10]. Under high salt conditions, we detected no significant interaction of either *Pae *or *Eco*χψ with EcoSSBQ152*, whereas *Pae*χψ interacted with wild-type *Eco*SSB with a similar affinity than with *Pae*SSB (data not shown). Since the 120 N-terminal amino acids of *Pae*SSB and *Eco*SSB show 79% homology and the last 7 amino acids are identical [32], this finding makes it very likely that the highly conserved C-terminus of *Pae*SSB is the only binding site for *Pae*χψ.

### *Pae*ψ shows DNA-binding activity

Since the SSB proteins involved in DNA replication form a tight complex with ssDNA, we examined whether *Pae*χψ not only binds to SSB but also directly to nucleic acids. We therefore tested the binding of *Pae*χψ to fluorescently labeled 50mer-dsDNA and 50mer-ssDNA in electrophoretic mobility shift assays (EMSA) under low salt conditions. Both substrates clearly show shifts with increasing concentrations of *Pae*χψ, indicating binding of the protein complex to the nucleic acids (Additional file 5: **Figure S5 *A ***and ***B***). However, ssDNA is bound with a somewhat higher affinity than dsDNA (Additional file 5: **Figure S5 *C***), suggesting that the complex seems to preferentially bind to ssDNA.

**Figure 4:**
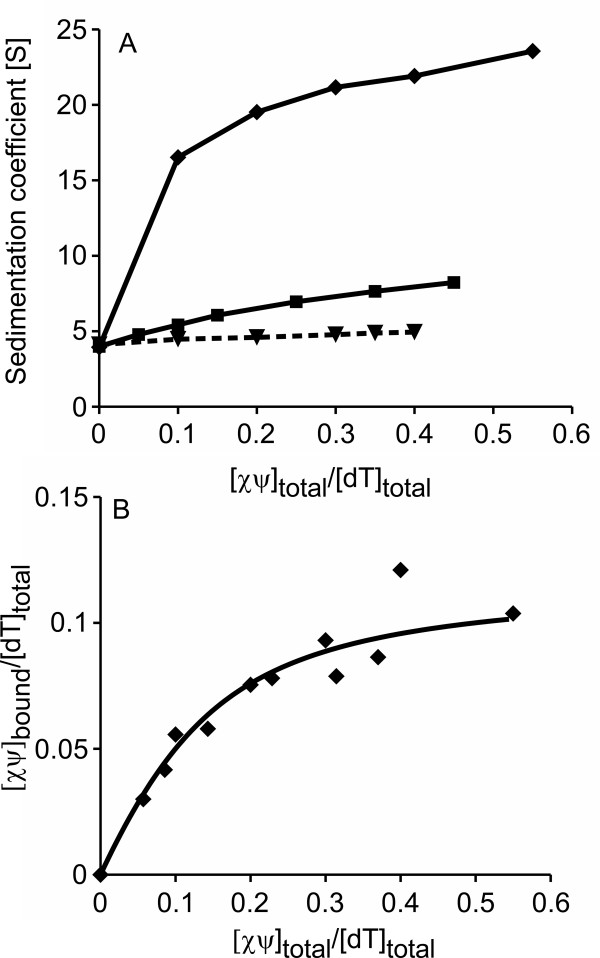
***Pae*χψ binds to ssDNA**. *(**A**) *Sedimentation coefficient of 35 μM poly(dT) in the presence of *Pae*χψ (*diamonds*), *Pae*χψ _(Δ1-85) _(*squares*) or *Eco*χψ (*triangles*) measured in low salt buffer at 20°C and 25000 rpm (λ = 280 nm). While the addition of *Pae*χψ increases the sedimentation coefficient of poly(dT) significantly, indicating the formation of a complex, *Eco*χψ shows only a slight effect. The binding of the truncated *Pae*χψ_(Δ1-85) _is severely reduced compared to wild-type, which shows that the N-terminus of *Pae*ψ is involved in ssDNA-binding. Lines are drawn just to guide the eye. *(**B**) *Binding isotherm for the *Pae*χψ/poly(dT) interaction, obtained by analysis of the *c*(*s*) distributions (see Methods section) of sedimentation velocity experiments at a (dT) concentration of 35 μM. The line represents a theoretical binding isotherm calculated for a simple interaction model of *n *molecules of χψ with one dT residue using the parameters: *n *= 0.12 and *K *= 5 × 10^5 ^M^-1^.

To further characterize the binding to ssDNA substrates, we then performed sedimentation velocity experiments in low salt buffer, titrating *Pae *or *Eco*χψ to a constant amount of poly(dT). The binding of the protein to ssDNA was monitored by an increase in the sedimentation coefficient of the latter. Whereas the addition of *Eco*χψ to poly(dT) did not significantly change the sedimentation coefficient of the ssDNA, addition of *Pae*χψ resulted in an increase from approximately 4 S to 24 S, indicating complex formation (Figure 4A). Therefore, in contrast to the *E. coli *complex, *Pae*χψ shows binding to ssDNA, which also supports the EMSA results.

To characterize this binding, the amount of free *Pae*χψ was determined from the *c*(*s*) distributions, and a binding isotherm was constructed (Figure 4B). The binding stoichiometry, defined as the amount of χψ bound per dT residue, was found to be 0.12. Therefore, one *Pae*χψ complex is able to bind about eight dT residues with a binding affinity of approximately 5 × 10^5 ^M^-1^. It is worth noting, however, that the binding affinity of *Pae*χψ to ssDNA is salt-dependant, since it was weakened at increasing salt concentrations, and no binding was observed in high salt buffer (data not shown). In this context, it is interesting to note that under low salt conditions, but not at elevated salt, ssDNA increases the affinity of *Eco*χ to *Eco*SSB about 20-fold [10]. Under elevated salt conditions, however, in addition to ssDNA, the presence of all other clamp loader subunits, which themselves do not significantly interact with the SSB/ssDNA complex, is required to increase the SSB-binding affinity of *Eco*χψ about 1000-fold [30]. This might be mediated by a conformational change in the N-terminus of ψ which accompanies the assembly of χψ into the clamp loader complex [16]. A similar conformational change is expected to occur in the conserved ultimate N-terminus of *Pae *ψ, which might also increase the ssDNA-binding affinity of *Pae*χψ under elevated salt conditions.

To determine which protein within the *Pae*χψ heterodimer is responsible for ssDNA-binding, we wanted to investigate the interaction of poly(dT) with only *Pae*χ or only *Pae*ψ. However, both proteins were insoluble if expressed on their own in *E. coli *and could not be purified. Therefore, we tried to produce the chimeric complexes χ*_Eco_*ψ*_Pae _*and χ*_Pae_*ψ*_Eco_*, but only *Eco*χ and *Pae*ψ formed a soluble complex. Although these two proteins are from different organisms, they formed a stable homogeneous heterodimeric χ*_Eco_*ψ*_Pae _*complex as revealed by sedimentation velocity experiments (data not shown). This is most probably due to the fact that the residues at the χψ interface are conserved between *E. coli *and *P. aeruginosa *[18]. Similarly to *Pae*χψ, χ*_Eco_*ψ*_Pae _*is able to bind to poly(dT) under low salt conditions (Additional file 6). Since we showed that *Eco*χψ does not bind ssDNA (Figure 4A), and under similar conditions no direct interaction between *Eco*χ and ssDNA could be detected [9,10,30,34], it has to be the ψ subunit within *Pae*χψ which binds to ssDNA.

To check whether the apparently unstructured N-terminal region of *Pae*χψ is involved in the interaction with ssDNA, we tested the binding of the truncated *Pae*χψ_(Δ1-85) _complex to poly(dT). As can be seen by the smaller increase in the sedimentation coefficient of the protein/ssDNA complex compared to full-length *Pae*χψ (Figure 4A), less *Pae*χψ_(Δ1-85) _can bind to ssDNA. Therefore, the N-terminal region of ψ is involved in ssDNA-binding.

### *P. putida *χψ also binds to ssDNA

When the ψ subunit of *P. aeruginosa *was discovered [18], a whole family of ψ proteins present within several species of the Pseudomonadaceae was identified based on sequence comparisons. To test whether ssDNA-binding is a general property of the pseudomonal ψ proteins, we cloned and purified the χψ complex from *P. putida*. In sedimentation velocity experiments with poly(dT) in low salt buffer, *P. putida *(*Ppu*) χψ, similarly to *Pae*χψ, increased the sedimentation coefficient of poly(dT), indicating ssDNA-binding (Additional file 6).

### *Pae*χψ binds to ssDNA covered by *Eco*SSB+Gly

The ssDNA at the replication fork is entirely covered by SSB [31,35]. To have an *in vitro *setting which most resembles the *in vivo *situation, the binding of *Pae*χψ to ssDNA complexed with SSB was analyzed. As this experiment is complicated by the fact that χψ also binds to SSB, we used a C-terminal extension mutant of *Eco*SSB, *Eco*SSB+Gly. This protein carries an additional glycine residue at its C-terminus which dramatically weakens its interaction with *Eco*χ [15]. Using sedimentation velocity experiments, we first confirmed that the binding of both *Pae *and *Eco*χψ to this mutant is drastically reduced (Additional file 7).

**Figure 5:**
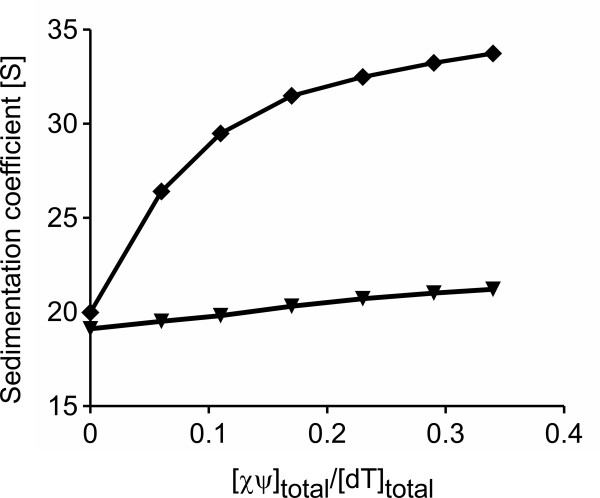
***Pae*χψ interacts with ssDNA that is covered by *Eco*SSB+Gly**. *Pae*χψ (*diamonds*) or *Eco*χψ (*triangles*) were titrated to a saturated *Eco*SSB+Gly/poly(dT) complex containing 1 μM SSB and 35 μM dT in low salt buffer. The mixtures were sedimented at 22000 rpm and 20°C, with λ = 280 nm. Lines are drawn just to guide the eye. Whereas the addition of *Eco*χψ has only a slight effect that might be due to the binding of *Eco*χψ to *Eco*SSB+Gly, *Pae*χψ increases the sedimentation coefficient from 20 S to about 34 S, indicating that *Pae*χψ is able to bind to ssDNA even when the latter is saturated with SSB proteins.

To test whether χψ can interact with ssDNA even when ssDNA is bound by SSB, a saturated complex of *Eco*SSB+Gly and poly(dT) (35 dT residues per tetramer) was titrated with *Pae *or *Eco*χψ in low salt buffer. When the *Eco*SSB+Gly/ssDNA complex was analyzed alone, it showed a sedimentation coefficient of approximately 19 S (Figure 5). The addition of *Eco*χψ increased the sedimentation coefficient only slightly, to about 21 S, which could be due to a weak binding of *Eco*χψ to *Eco*SSB+Gly. The addition of *Pae*χψ, however, increased the sedimentation coefficient from approximately 20 S to 34 S. This clearly shows the formation of a large ternary complex, indicating that *Pae*χψ is able to bind to ssDNA even when ssDNA is covered by SSB.

### *Pae*χψ binds with a higher affinity to SSB/ssDNA complexes than *Eco*χψ

**Figure 6:**
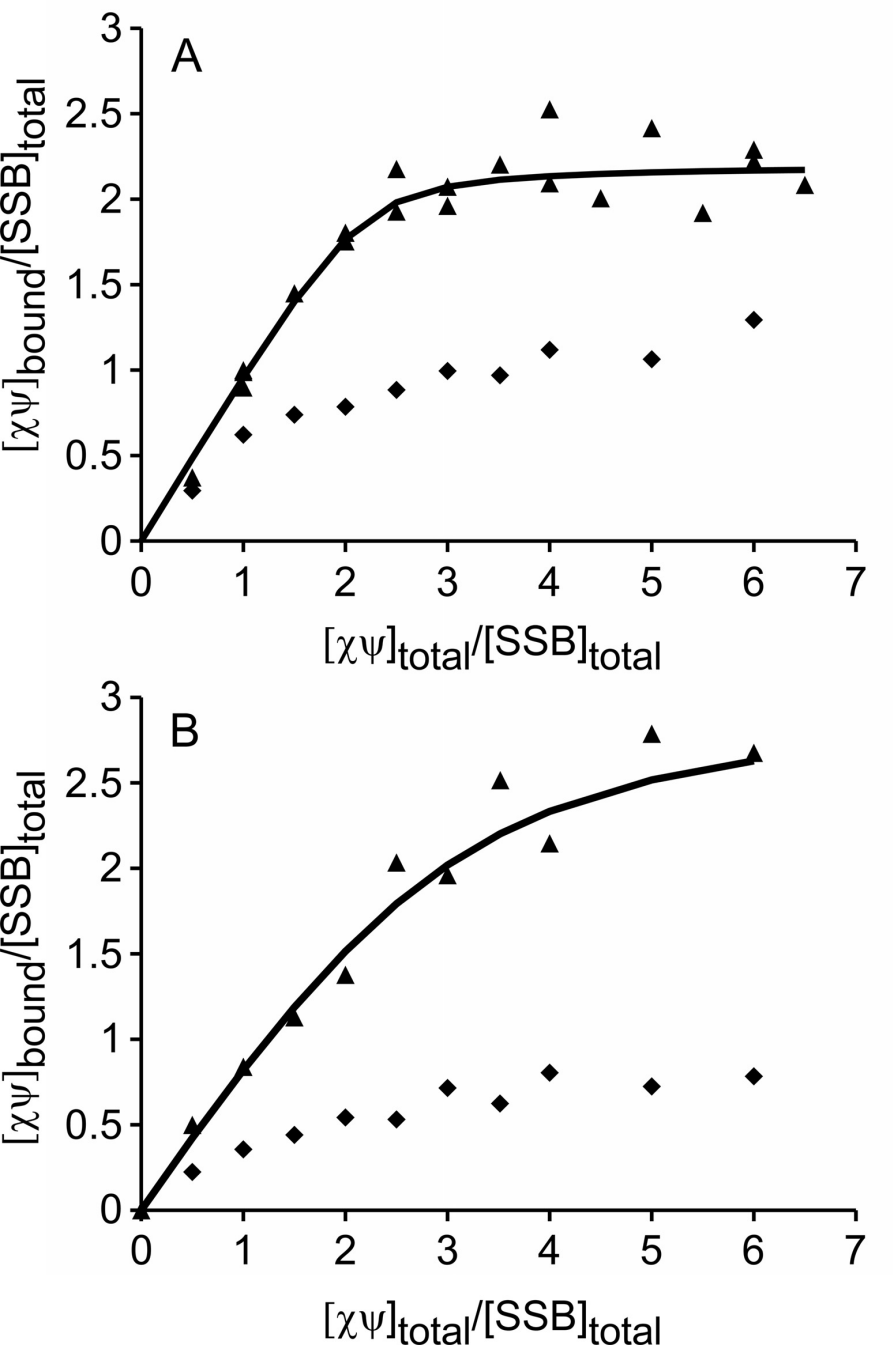
***Pae*χψ binds to an SSB/ssDNA complex with higher affinity than *Eco*χψ**. *(**A**) Pae*χψ or *(**B**) Eco*χψ were titrated to 0.35 μM of their respective SSB in absence (*diamonds*) or presence (*triangles*) of 12.3 μM poly(dT), in low salt buffer (50000 rpm or 22000 rpm in the absence or presence of poly(dT), respectively, 20°C, λ = 230 nm). The lines represent theoretical binding isotherms calculated for a simple interaction model of *n *molecules of χψ with one SSB tetramer using the following parameters: *(**A**) n *= 2.2, *K *= 5 × 10^7 ^M^-1 ^and *(**B**) n *= 3, *K *= 6 × 10^6 ^M^-1^. The affinity of *Pae*χψ for the SSB/ssDNA complex is so high that only a lower limit of the binding constant can be given.

To test whether the ability of *Pae*χψ to bind both *Pae*SSB and ssDNA results in an increased affinity toward SSB/ssDNA complexes, its binding to *Pae*SSB in the presence or absence of poly(dT) was examined in low salt buffer, and the results were compared to the *E. coli *system. For this purpose, 0.35 μM of the respective SSB protein, with or without saturating amounts of poly(dT), were titrated with *Pae *or *Eco*χψ, and were analyzed in sedimentation velocity experiments. Figure 6 shows that the binding affinity of both *Pae*χψ and *Eco*χψ toward the SSB/poly(dT) complex is significantly enhanced compared to the affinity toward free SSB protein, which is consistent with the observation that under low salt conditions, the affinity of *Eco*χ to *Eco*SSB is increased about 20-fold in the presence of ssDNA [10]. This is most probably due to a conformational change of SSB induced by the binding to long stretches of ssDNA, making the highly conserved C-terminal region more easily accessible for interaction with other proteins [36].

In the absence of poly(dT), the binding was too weak to determine the binding parameters for either *P. aeruginosa *or *E. coli *(Figure 6). Due to the aggregation of *Pae*χψ at higher protein concentrations at low salt, the binding could not be examined at concentrations which would have allowed for the determination of the binding parameters. The binding of *Pae*χψ to the SSB/ssDNA complex, however, is so strong that even at the lowest concentration that can be applied in AUC experiments using the absorbance optics, stoichiometric binding was observed and the affinity is larger than 5 × 10^7 ^M^-1 ^(Figure 6A). Therefore, the affinity of *Pae*χψ to SSB/poly(dT) is at least eight times higher than the affinity of the respective *E. coli *proteins (K= 6 × 10^6 ^M^-1^, Figure 6B). This is most probably a result of the ssDNA-binding affinity of *Pae*ψ which enhances the stability of the ternary complex, when compared to that of the *E. coli *system.

## Conclusions

The χ and ψ subunits are part of the clamp loader of DNA polymerase III holoenzyme. Whereas *Eco*χ and *Pae*χ have a similar size and show about 40% sequence homology, *Pae*ψ is nearly twice as large as its *E. coli *counterpart. The N-terminal residues of ψ that are needed for the interaction with the minimal clamp loader [16] and the residues involved in binding χ [14] are conserved, however *Pae*ψ contains additional sequences that are absent from the *E. coli *protein. We showed by homology-based modeling that these additional sequences do not interfere with secondary structure elements of *Eco*ψ, but can be assigned to loop regions of the protein (Figure 2B).

Analytical ultracentrifugation experiments revealed that *Pae*χψ has a higher degree of asymmetry than its *E. coli *counterpart. This asymmetry is reduced by the truncation of the 85 N-terminal amino acids of *Pae*ψ. SAXS experiments of *Pae*χψ and of this truncated variant, as well as molecular modeling, strongly support the idea that the N-terminus of *Pae*ψ is elongated and probably highly flexible. Our results show that this N-terminus is responsible for ssDNA-binding of *Pae*χψ under low salt conditions, a property which can not be found in *Eco*χψ.

Additionally, we could show that also *P. putida *χψ is able to bind to ssDNA. To our knowledge, this is the first report on the DNA-binding of ψ subunits of DNA polymerase III. Homology searches within the Pseudomonadaceae revealed several proteins related to *Pae*ψ, all containing extra sequences absent from *Eco*ψ, which were in many cases highly conserved [18]. One conserved region is located in the unstructured N-terminus of ψ, includes several positively charged amino acids and might be responsible for the binding of the pseudomonal ψ proteins to DNA (Additional file 8).

Using an *Eco*SSB mutant impaired in χψ binding, we showed that *Pae*χψ is even able to bind to ssDNA that is covered with SSB. Furthermore, we demonstrated that the affinity of *Pae*χψ to SSB/ssDNA complexes is significantly increased compared to the *E. coli *system. This fact is most probably due to the ability of *Pae*ψ to bind to ssDNA. It has been shown that under low salt conditions only *Pae*χψ has a significant influence on DNA synthesis by its cognate DNA polymerase III [17,18]. The addition of *Pae*χψ and SSB shows a synergistic effect, increasing the activity of the minimal clamp loader *Pae*τ_3_δδ' 25-fold, whereas *Eco*τ_3_δδ' is insensitive to the addition of *Eco*χψ [17,18]. Even though it might not be the only mechanism, the binding of *Pae*ψ to DNA very likely contributes to the higher stimulatory effect of *Pae*χψ on the minimal clamp loader in the presence of SSB. Since we showed that the χψ complex of *P. putida *is also able to bind DNA, this stimulatory effect might be a characteristic of pseudomonal χψ complexes.

In spite of the fundamental aspects of this work, the full understanding of the DNA polymerase III holoenzyme of *P. aeruginosa*, and of its ψ subunit in particular, may reveal new targets for the development of specific inhibitors of pseudomonal DNA replication. The most striking difference between the *E. coli *and pseudomonal χψ complexes identified in this work is the ssDNA-binding ability mediated by the N-terminus of *Pae*ψ. Since we showed that this region is unstructured, it can probably not be used directly in antipseudomonal drug design. However, it is tempting to speculate that this region might fold upon ssDNA-binding and one of our future objectives is to solve the structure of a *Pae*χψ/ssDNA complex.

## Methods

### Buffers and reagents

Poly(dT) (~1400 nt in length) was purchased from GE Healthcare Life Sciences. Its concentration is given in monomer residues throughout the text and was determined spectrophotometrically, using an absorption coefficient of 8600 M^-1 ^cm^-1 ^at maximum [37]. Protein concentrations were determined spectrophotometrically, using the absorption coefficients at 280 nm calculated from amino acid composition [38]: *Pae*SSB: 95800 M^-1 ^cm^-1^; *Pae*χψ: 51450 M^-1 ^cm^-1^; *Pae*χψ_(Δ1-85)_: 45950 M^-1 ^cm^-1^; *Ppu*χψ: 55920 M^-1 ^cm^-1^; *Eco*χψ: 52940 M^-1 ^cm^-1^; chimeric χ*_Eco_*ψ*_Pae_*: 63940 M^-1 ^cm^-1^. For wild-type *Eco*SSB and *Eco*SSB+Gly an absorption coefficient of 113000 M^-1 ^cm^-1 ^was used [39].

Experiments were carried out in potassium phosphate buffer (KP_i_) pH 7.4, containing two different NaCl concentrations: high salt buffer (0.3 M NaCl, 20 mM KP_i _pH 7.4, 0.5 mM DTT), and low salt buffer (5 mM NaCl, 5 mM KP_i _pH 7.4, 0.87 M glycerol, 0.5 mM DTT).

### Cloning of the χ and ψ genes of *P. aeruginosa*, *E. coli*, and *P. putida*

Primer sequences are given in additional file 9. The *holC *and *holD *genes of *P. aeruginosa *were amplified by PCR from genomic DNA of strain PAO1. For the *holD *gene, the start codon was at the position identified in [18]. After *Bsa*I/*Bam*HI digestion, the *holC *PCR product was cloned into pCDFDuet-1 (Novagen), cut with *Nco*I/*Bam*HI. The *holD *PCR product was cut with *Bgl*II/*Eco*RI and cloned into the pGEX-6P-1 vector (GE Healthcare Life Sciences), digested with *Bam*HI/*Eco*RI.

The *holC *and *holD *genes of *E. coli *were amplified by PCR from genomic DNA of *E. coli *strain LK111λ [40]. The *holC *PCR product was cut with *Nco*I/*Xho*I, and cloned into pET-15b (Novagen), cut with the same enzymes. This construct was then digested with *Xho*I, and the *holD *PCR product, cut with *Xho*I, was ligated in.

For expression of the chimeric χ*_Eco_*ψ*_Pae _*complex, the *holC *gene was amplified by PCR from genomic DNA of *E. coli *LK111λ. After digestion with *Nde*I/*Xho*I, the PCR product was cloned into the vector pCDFDuet-1.

The *holC *and *holD *genes of *P. putida *were amplified by PCR from genomic DNA of *P. putida *strain KT2440 [41] and were cloned into the vectors pRSFDuet-1 (Novagen) and pETGEX-6P, respectively. To construct the latter, the sequence coding for GST and the multiple cloning site were amplified from pGEX-6P-1, and were cloned into pET-15b. In the resulting vector, protein expression is under control of the T7*lac *promoter and is therefore more tightly regulated than in pGEX-6P-1. For the *holD *gene, the start codon was at the position identified in [18]. After digestion with *Bsa*I/*Bam*HI, the *holC *PCR product was cloned into pRSFDuet-1, cut with *Nco*I/*Bam*HI. The *holD *PCR product was cut with *Bam*HI/*Xho*I and ligated into pETGEX-6P.

All constructs were checked for errors by sequencing the complete genes (GATC Biotech).

### Construction of the N-terminally truncated mutant *Pae*ψ_(Δ1-85)_

A 582 bp fragment, encoding *Pae*ψ truncated of its first 85 amino acids (*Pae*ψ_(Δ1-85)_), was amplified by PCR from the genomic DNA of *P. aeruginosa *strain PAO1 (for sequence of primers see additional file 9). After digestion with *Bgl*II/*Eco*RI, the PCR fragment was cloned into pGEX-6P-1, cut with *Bam*HI/*Eco*RI, allowing for the expression of N-terminally GST-tagged *Pae*ψ_(Δ1-85)_.

### Protein expression and purification

*Pae*χψ, *Pae*χψ_(Δ1-85)_, chimeric χ*_Eco _*ψ*_Pae _*and *Ppu*χψ, were expressed as χ/GSTψ. During the purification procedure the GST moiety was cleaved off by PreScission™ protease digestion. *Eco*χψ was expressed untagged. Expression of the χ proteins was done using Duet vectors (Novagen). While *Pae*ψ and *Pae*ψ_(Δ1-85) _were expressed using pGEX-6P-1, *Ppu*ψ was expressed from pETGEX-6P.

*E. coli *Rosetta (DE3) pLyS cells (Novagen) were transformed with the respective vectors encoding χ and GSTψ proteins and were grown to E_600 nm_= 1.2 in a 10 L fermentor flask at 37°C in LB medium. 5 h after addition of 1 mM IPTG cells were harvested, resuspended in an equal volume of PBS (300 mM NaCl, 2.7 mM KCl, 10 mM Na_2_HPO_4_, 1.8 mM KH_2_PO_4_, 10% (v/v) glycerol, 1 mM DTT, pH 7.3) and frozen in N_2 _(liq). 40 g of frozen cells were thawed in the presence of two volumes of PBS buffer, 1 mM EDTA, 1.67 mM DTT, 0.1 mM PMSF, and 10 tablets of complete EDTA-free protease inhibitor (Roche). The suspension was incubated for 20 min at 4°C with 0.3 mg/mL lysozyme, followed by sonification. After centrifugation, the supernatant was applied onto a glutathione sepharose 4B (GE Healthcare Life Sciences) column preequilibrated with PBS buffer. After washing with GS buffer (50 mM Tris-HCl pH 7.0, 150 mM NaCl, 10% glycerol (v/v), 1 mM EDTA, 1 mM DTT), the column was incubated overnight at 4°C with 840 μg of PreScission™ protease (GE Healthcare Life Sciences). The χ and ψ proteins were eluted using GS300 buffer (GS buffer with 0.3 M NaCl). Fractions containing the purest complex were pooled and the proteins were precipitated for 1 h with 164 g/L (NH_4_)_2_SO_4 _(except for *Pae*χψ_(Δ1-85) _where 300 g/L were used). After centrifugation, the precipitated proteins were resuspended in GS300 buffer and subjected to size exclusion chromatography (Superdex-75-prepgrade, GE Healthcare Life Sciences) in the same buffer. Fractions that contained contamination-free χψ complex were pooled, dialyzed overnight against high salt buffer containing 10% (v/v) glycerol, flash frozen in N_2 _(liq) and stored at -80°C.

Expression of the *Eco*χψ complex was done using the pET-15b vector (Novagen). For purification, cells harvested from the fermentor culture were resuspended in 50 mM Tris-HCl pH 7.5, 10% (w/v) sucrose and frozen in N_2 _(liq). 30 g of frozen cells were thawed in the presence of 0.1 mM PMSF and cell lysis was done as above, but using 0.1 mg/mL lysozyme. After centrifugation, the proteins in the supernatant were precipitated with 250 g/L (NH_4_)_2_SO_4 _at 4°C. The protein pellet after centrifugation was resuspended in buffer A20 (50 mM Tris pH 7.8, 20 mM NaCl, 10% (v/v) glycerol, 1 mM EDTA, 1 mM DTT), and dialyzed against the same buffer. The solution was applied onto a Q-sepharose fast flow (GE Healthcare Life Sciences) column preequilibrated in the same buffer. After washing with buffer A20, the proteins were eluted in a gradient of 20 mM to 250 mM NaCl in buffer A20. The fractions containing *Eco*χ and ψ were pooled and the protein complex was precipitated with 300 g/L of (NH_4_)_2_SO_4 _at 4°C. Following centrifugation, the protein pellet was resuspended in buffer A50 (A20 with 50 mM NaCl) and applied onto a size exclusion chromatography column (see above) preequilibrated with the same buffer. Fractions containing a contamination-free χψ complex were pooled and dialyzed overnight against 20 mM Hepes pH 8.0, 100 mM NaCl, 2 mM DTT, 1 mM NaN_3_, and 3% (w/v) sucrose, flash frozen in N_2 _(liq), and stored at -80°C.

Expression and purification of *Eco*SSB and *Eco*SSB+Gly was performed as described previously [15]. *Eco*SSB Q152* [33] and *Pae*SSB [32] were expressed and prepared as described earlier.

### Analytical ultracentrifugation

Analytical ultracentrifugation experiments were performed either in a Beckman Optima XL-A ultracentrifuge or a Beckman/Coulter ProteomeLab XL-I ultracentrifuge, using An-50 Ti rotors. Concentration profiles were measured with the UV-absorption scanning optics of the centrifuge.

Sedimentation velocity experiments were carried out at 20°C in either 12 mm or 3 mm standard double-sector centerpieces filled with 400 μL or 100 μL sample, respectively, at the indicated rotor speeds. For the analysis of protein-protein or protein-DNA interactions, the slower sedimenting molecule was titrated to a constant concentration of the faster sedimenting one. Since the reactions governing the interactions examined in this study are fast compared to the timescale of sedimentation, only two sedimenting boundaries are observed [42]. The slow one represents the free slower sedimenting molecule, whereas the fast one contains complexes of both components and free faster sedimenting molecules.

The measured concentration profiles were evaluated using SEDFIT [19] which transforms them into diffusion-corrected sedimentation coefficient distributions [*c*(*s*) distributions]. As the areas under the separate peaks in the *c*(*s*) distributions are a measure of the absorbance of the species represented by the peaks [42], this information can be used to determine binding isotherms [29], which were evaluated using a simple interaction model as described previously [10].

In the case of the χψ/SSB/poly(dT) experiments where a wavelength of 230 nm was used, the extinction coefficients of χψ and the SSB/poly(dT) complexes were determined from the observed absorbances in the centrifuge and the known initial concentrations of the components. Thus, it was possible to correct for variations in the exact wavelength provided by the monochromator of the centrifuge.

For hydrodynamic analysis, measured s-values were corrected to s_20, W_, using the partial specific volumes calculated from amino acid composition [43]. Since the partial specific volume of complexes of different macromolecules with unknown composition can not be calculated, uncorrected sedimentation coefficients are given in these cases.

Sedimentation equilibrium experiments were carried out at 4°C in either 12 mm or 3 mm double-sector centerpieces filled with 150 μL or 40 μL sample, respectively. Absorbance was detected at 280 nm and rotor speeds of 9000, 13000 and 18000 rpm were used. Analysis of the data was performed as described earlier [44].

### SAXS measurements and modeling of *Pae*ψ

Sample preparation for SAXS included dialysis in 20 mM Hepes pH 7.4, 50 mM NaCl, 3% (w/v) sucrose, 1 mM NaN_3_, 1 mM DTT, followed by protein concentration using Vivaspin centrifugal concentrators (Sartorius Stedim Biotech). The flowthrough was taken as buffer reference in SAXS data collection. SAXS data were collected at EMBL/DESY X33 beamline (Hamburg, Germany) at 20°C cell temperature. Molar mass of the samples was determined by comparison of scattering intensity at zero angle I(0) obtained from Guinier analysis (s*R_G _< 1.3 in ln(I) vs. s^2^-plot) with reference proteins BSA and lysozyme, and was additionally determined from volume (Porod volume, see e.g. [23]).

SAXS data were processed and analyzed using the ATSAS package [45]. *Pae*χψ and *Pae*χψ_(Δ1-85) _were measured at protein concentrations of 2, 4 and 8 mg/ml and 1.3, 3.2 and 6.4 mg/ml, respectively. All sample scattering curves were corrected by subtraction of the corresponding buffer scattering curves. Scattering curves were merged as described in e.g. [23] to obtain optimal data quality at lower and higher s-ranges. For full-length *Pae*χψ, a flexible modeling approach was performed, using the ensemble optimization method (EOM) described in e.g. [26,27]. For the truncated *Pae*χψ_(Δ1-85) _construct, an independent set of ten *ab initio *bead models was calculated with GASBOR without prior symmetry information or other restrictions. Models were aligned and averaged using DAMAVER. For better representation, an electron density envelope of the averaged bead model was calculated using the SITUS package [46]. SAXS-shape representation and docking into the SAXS shape were performed with the UCSF chimera package [47] and the superposition of structures was done using PyMOL [48].

Modeling of the *Pae*ψ molecule was performed using the Bioinformatic Toolkit server [24]. The *Pae*ψ sequence was first analyzed by HHpred [49], identifying *Eco*ψ (pdb code: 1em8) as one of the best candidates with highest score (probability 96.66, E-value= 0.02, Identities 21%). This structure was then chosen as a template for MODELLER [25].

### Electrophoretic mobility shift assays (EMSA)

EMSA was performed in 5% polyacrylamide gels in Tris-borate buffer (running buffer: 45 mM Tris, 45 mM boric acid; gels: 25 mM Tris, 25 mM boric acid) using fluorescently FAM- labeled oligonucleotides (Thermo Fischer). Gels were analyzed using a Typhoon Scanner (488 nm laser, GE Healthcare Life Sciences), and quantified using the ImageQuant software (GE Healthcare Life Sciences). 1 μl of DNA was added to the protein in 9 μl low salt buffer to yield the indicated final concentrations. The mixture was incubated for 10 min at room temperature prior to gel electrophoresis at 5°C. Double-stranded DNA was prepared by annealing of oligonucleotides A and B; oligonucleotide A was used as the ssDNA sample (A: 5'6FAM-GGATACGTAACAACGCTTATGCATCGCCGCCGCTACATCCCTGAGCTGAC 3'. B: 5'GTCAGCTCAGGGATGTAGCGGCGGCGATGCATAAGCGTTGTTACGTATCC 3').

## Conflict of interests statement

The authors declare that they have no competing interests.

## Authors' contributions

Conceived and designed the experiments: SEHM, GW, UC. Performed the experiments: SEHM, GW, NN. Analyzed the data and wrote the manuscript: SEHM, GW, NN, UC. All authors read and approved the final manuscript.

## Supplementary Material

Additional file 1 **Figure S1**. Sedimentation equilibrium concentration gradients of *Pae*χψ and *Eco*χψ. Click here for file

Additional file 2 **Figure S2**. *c*(*s*) distributions for *Pae*χψ, *Eco*χψ and *Pae*χψ_(Δ1-85)_. Click here for file

Additional file 3 **Figure S3**. SAXS data of *Pae*χψ and *Pae*χψ_(Δ1-85)_. Click here for file

Additional file 4 **Figure S4**. Kratky-plot (I·s^2 ^vs. s) of the full-length *Pae*χψ and the truncated form *Pae*χψ_(Δ1-85) _scattering data. Click here for file

Additional file 5 **Figure S5**. Electrophoretic mobility shift assays of *Pae*χψ with fluorescently labeled ssDNA and dsDNA. Click here for file

Additional file 6 **Figure S6**. The chimeric complex χ*_Eco_*ψ*_Pae _*and *Ppu*χψ interact with ssDNA. Click here for file

Additional file 7 **Figure S7**. The binding of χψ to the C-terminal extension mutant *Eco*SSB+Gly is severely reduced. Click here for file

Additional file 8 **Figure S8**. Sequence alignment of ψ proteins from different members of the Pseudomonadaceae. Click here for file

Additional file 9 **Primer sequences used for cloning of the χ and ψ genes of *P. aeruginosa*, *E. coli *and *P. putida***. Click here for file
