# Supplementary material for: Characterization of the χψ subcomplex of Pseudomonas aeruginosa DNA polymerase III
Source: BMC Mol Biol. 2011 Sep 28;12:43. doi: 10.1186/1471-2199-12-43 (PMC3197488; doi:10.1186/1471-2199-12-43)
Supplement: Additional file 1 — Figure S1. Sedimentation equilibrium concentration gradients of Paeχψ and Ecoχψ. [file 1471-2199-12-43-S1.PDF]

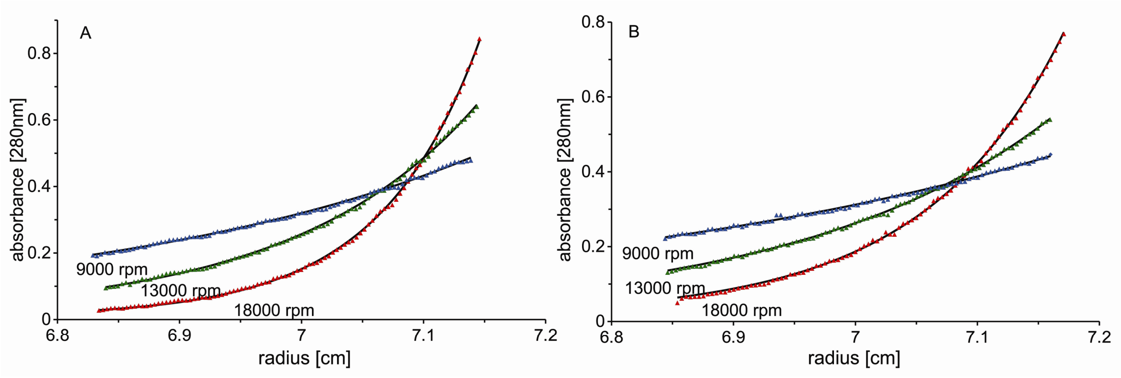

Figure S1. Sedimentation equilibrium concentration gradients of *(A)* 5.8 µM *Pae*χψ and *(B)* 5.7 µM *Eco*χψ. The experiments were done at three different speeds (*red*: 18000 rpm; *green*: 13000 rpm; and *blue*: 9000 rpm) and 4°C, in high salt buffer (λ=280 nm). The points represent the measured data and the black lines represent the results of global fitting with a model of a single species of ***(A)*** 46 (± 4) kg/mol and ***(B)*** 31 (± 4) kg/mol. Therefore, both *Pae*χψ and *Eco*χψ exist as heterodimers in solution.
