# Supplementary material for: Characterization of the χψ subcomplex of Pseudomonas aeruginosa DNA polymerase III
Source: BMC Mol Biol. 2011 Sep 28;12:43. doi: 10.1186/1471-2199-12-43 (PMC3197488; doi:10.1186/1471-2199-12-43)
Supplement: Additional file 2 — Figure S2. c(s) distributions for Paeχψ, Ecoχψ and Paeχψ(Δ1-85). [file 1471-2199-12-43-S2.PDF]

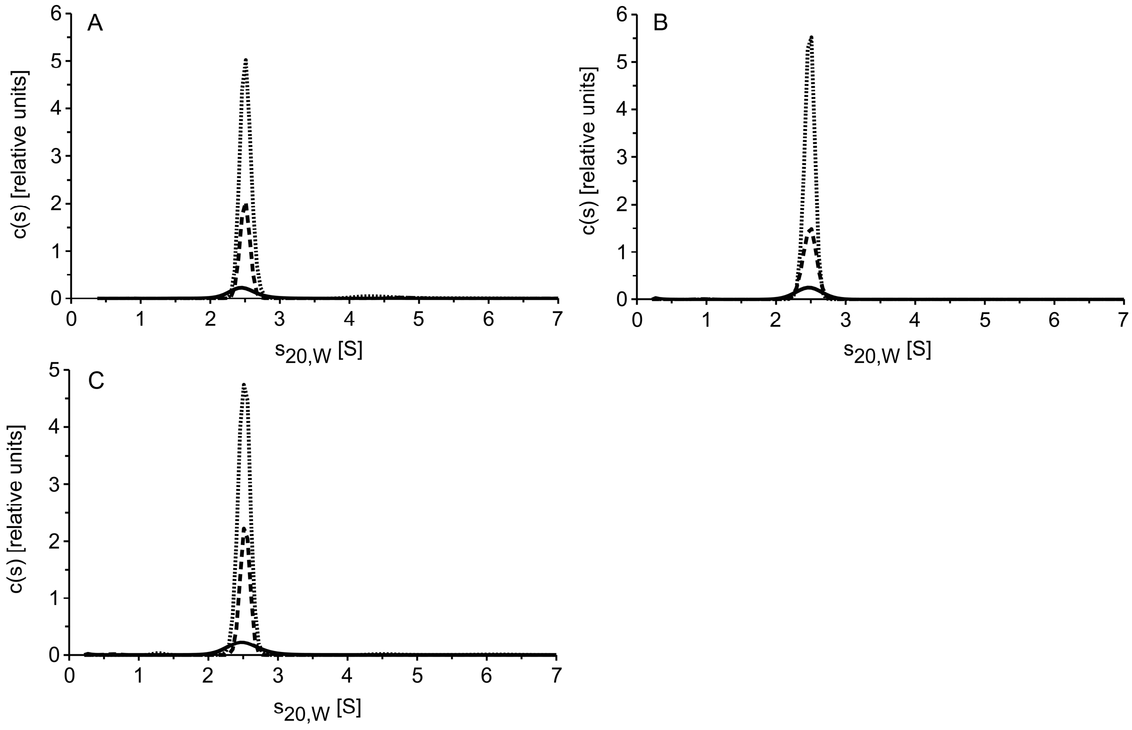

Figure S2. *c*(*s*) distributions for three concentrations of ***(A)*** *Paeχψ* (1.9 µM: *solid line*; 5.5 µM: *dashed line*; and 15.5 µM: *fine dashed line*), ***(B)*** *Ecoχψ* (1.9 µM: *solid line*; 5.7 µM: *dashed line*; and 15.1 µM: *fine dashed line*) and ***(C)*** *Paeχψ*$_{(\Delta 1\text{-}85)}$ (2.2 µM: *solid line*; 6.5 µM: *dashed line*; and 17.4 µM: *fine dashed line*). The proteins were sedimented in high salt buffer at 50000 rpm (20°C, λ=280 nm). The single peak indicates that the protein preparations are homogeneous and contain no significant impurities or aggregation, and that in this concentration range a stable complex is formed.
