# Supplementary material for: Characterization of the χψ subcomplex of Pseudomonas aeruginosa DNA polymerase III
Source: BMC Mol Biol. 2011 Sep 28;12:43. doi: 10.1186/1471-2199-12-43 (PMC3197488; doi:10.1186/1471-2199-12-43)
Supplement: Additional file 3 — Figure S3. SAXS data of Paeχψ and Paeχψ(Δ1-85). [file 1471-2199-12-43-S3.PDF]

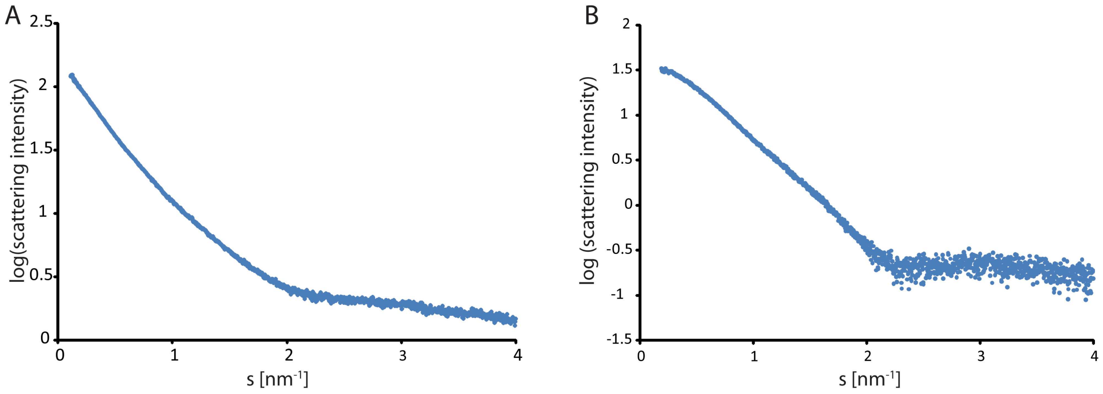

Figure S3. SAXS data. ***(A)*** Scattering profile of *Pae*χψ in buffer containing 20 mM Hepes pH 7.4, 50 mM NaCl, 3% (w/v) sucrose, 1 mM NaN_3, 1 mM DTT. ***(B)*** Scattering profile of the N-terminally truncated *Pae*χ$\psi_{(\Delta 1\text{-}85)}$ in the same buffer.
