# Supplementary material for: Characterization of the χψ subcomplex of Pseudomonas aeruginosa DNA polymerase III
Source: BMC Mol Biol. 2011 Sep 28;12:43. doi: 10.1186/1471-2199-12-43 (PMC3197488; doi:10.1186/1471-2199-12-43)
Supplement: Additional file 4 — Figure S4. Kratky-plot (I·s2 vs. s) of the full-length Paeχψ and the truncated form Paeχψ(Δ1-85) scattering data. [file 1471-2199-12-43-S4.PDF]

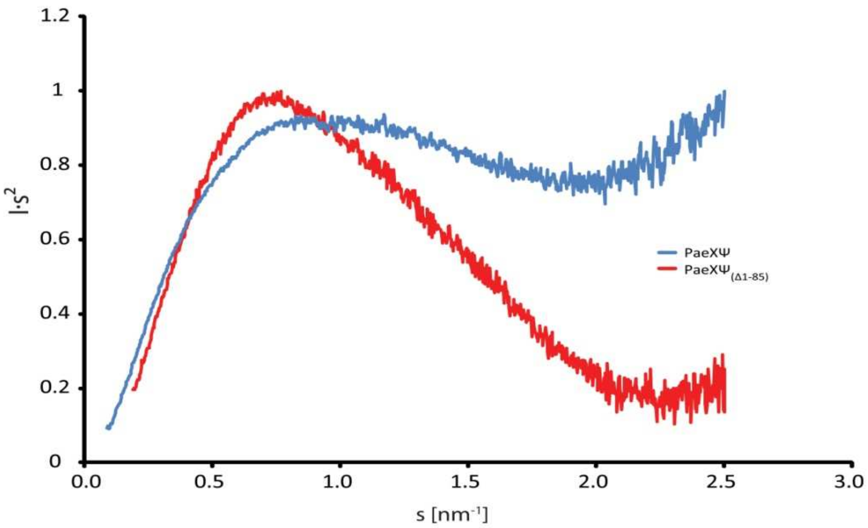

Figure S4. Kratky-plot (I·s² vs. s) of the full-length *Paeχψ* (*blue*) and the truncated form *Paeχψ*$_{(\Delta 1\text{-}85)}$ (*red*) scattering data. The bell-shaped curve of the shortened construct indicates that the protein is folded [1,2]. In contrast, the full-length protein is obviously flexible or unfolded to a large extent. Based on our modeling approach, more than 150 residues of *Paeψ* are either located in the flexible N-terminus or in loop-regions, which in total comprise more than 35% of the entire *Paeχψ* heterodimer, and thus are expected to have a dramatic effect on the shape of the Kratky-plot.

1. Putnam CD, Hammel M, Hura GL, Tainer JA: **X-ray solution scattering (SAXS) combined with crystallography and computation: defining accurate macromolecular structures, conformations and assemblies in solution.** Q Rev Biophys 2007, **40**(3):191-285.

2. Mertens HD, Svergun DI: **Structural characterization of proteins and complexes using small-angle X-ray solution scattering.** J Struct Biol 2010, **172**(1):128-141.
