# Supplementary material for: Characterization of the χψ subcomplex of Pseudomonas aeruginosa DNA polymerase III
Source: BMC Mol Biol. 2011 Sep 28;12:43. doi: 10.1186/1471-2199-12-43 (PMC3197488; doi:10.1186/1471-2199-12-43)
Supplement: Additional file 5 — Figure S5. Electrophoretic mobility shift assays of Paeχψ with fluorescently labeled ssDNA and dsDNA. [file 1471-2199-12-43-S5.PDF]

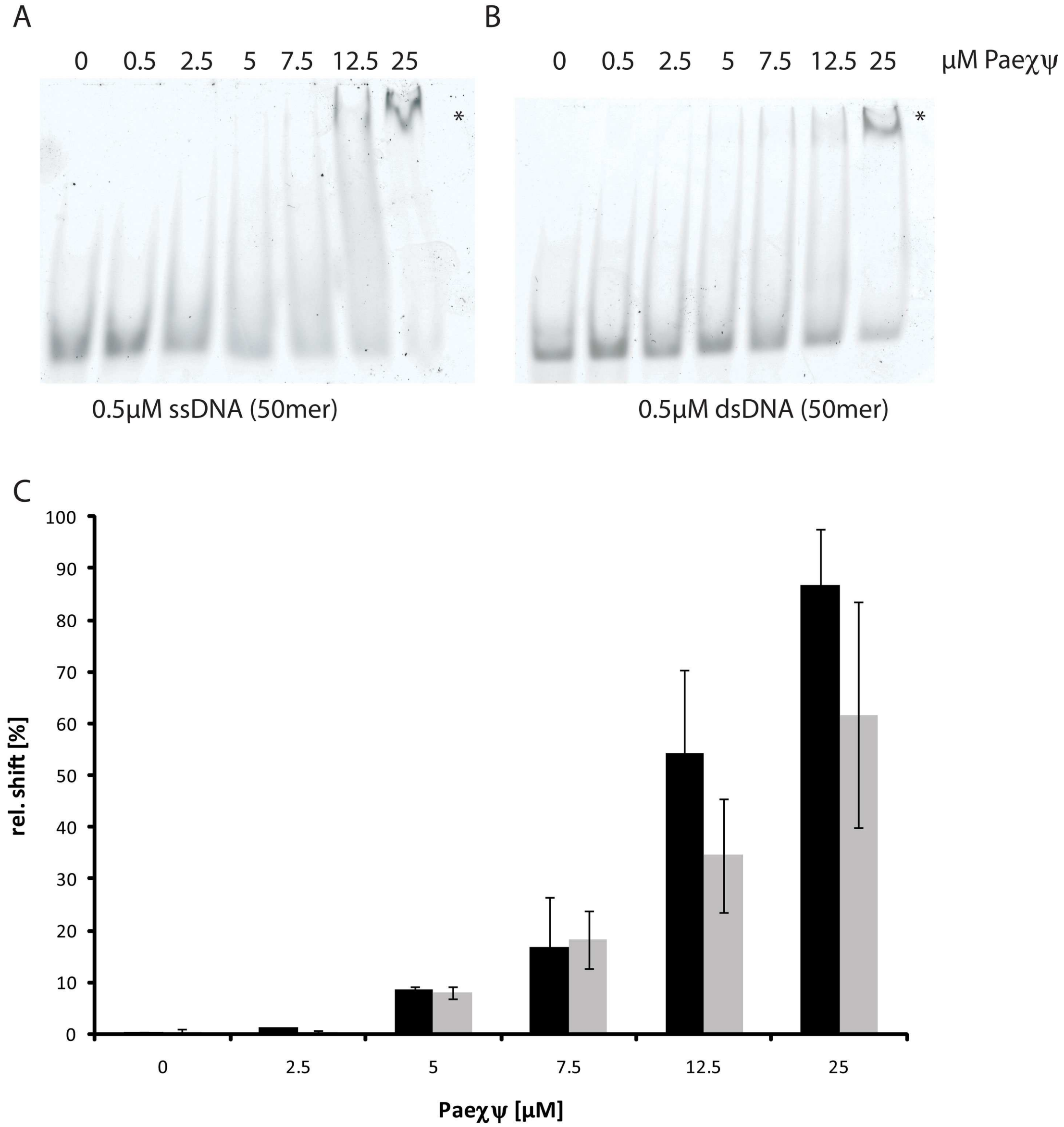

Figure S5. Electrophoretic mobility shift assays of *Paeχψ* with fluorescently (6FAM) labeled 50mer ssDNA *(A)* and dsDNA *(B)* in 5% polyacrylamide gels in Tris-borate buffer. Samples were incubated in low salt buffer for 10 min prior to gel electrophoresis. The asterisks indicate the *Paeχψ*:DNA complexes. ***(C)*** Quantitative analysis of multiple experiments as shown in ***(A)*** and ***(B)*** with standard deviations. *Paeχψ* binds to both DNA substrates but comparison shows that ssDNA (black bars) is bound more strongly than dsDNA (grey bars).
