# Supplementary material for: Characterization of the χψ subcomplex of Pseudomonas aeruginosa DNA polymerase III
Source: BMC Mol Biol. 2011 Sep 28;12:43. doi: 10.1186/1471-2199-12-43 (PMC3197488; doi:10.1186/1471-2199-12-43)
Supplement: Additional file 6 — Figure S6. The chimeric complex χEcoψPae and Ppuχψ interact with ssDNA. [file 1471-2199-12-43-S6.PDF]

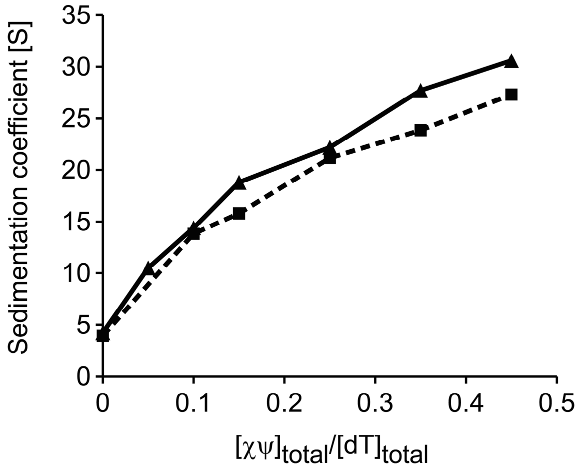

Figure S6. The chimeric complex $\chi_{Eco}\psi_{Pae}$ (*triangles*) and *Ppu*χψ (*squares*) interact with ssDNA. The proteins were titrated to 35 μM of poly(dT) in standard low salt buffer, and the mixtures were analyzed at 25000 rpm and 20°C (λ=280 nm) in an analytical ultracentrifuge. The addition of either χψ complexes increased the sedimentation coefficient of poly(dT) significantly. Lines are drawn just to guide the eye.
