# Supplementary material for: Characterization of the χψ subcomplex of Pseudomonas aeruginosa DNA polymerase III
Source: BMC Mol Biol. 2011 Sep 28;12:43. doi: 10.1186/1471-2199-12-43 (PMC3197488; doi:10.1186/1471-2199-12-43)
Supplement: Additional file 7 — Figure S7. The binding of χψ to the C-terminal extension mutant EcoSSB+Gly is severely reduced. [file 1471-2199-12-43-S7.PDF]

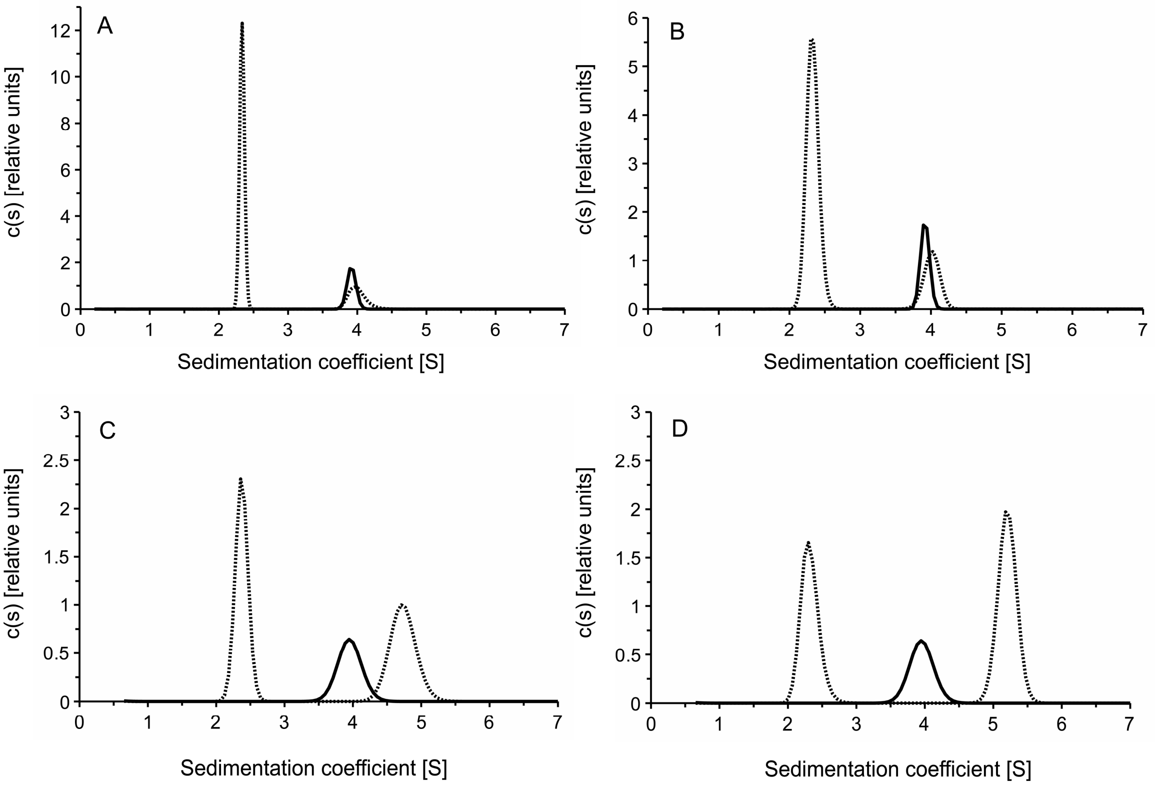

Figure S7. The binding of χψ to the C-terminal extension mutant *Eco*SSB+Gly is severely reduced. The *c*(*s*) distributions for 2.2 µM *Eco*SSB+Gly in the absence (*solid line*) or presence (*dashed line*) of a nine-fold molar excess of ***(A)** Paeχψ* or ***(B)** Ecoχψ* show that the sedimentation coefficient increases only slightly, by less than 0.15 S. This indicates a very weak binding of the χψ complexes to the SSB extension mutant. In contrast, the *c*(*s*) distributions for 2.2 µM *Eco*SSB wild-type in the absence (*solid line*) or presence (*dashed line*) of a seven-fold molar excess of ***(C)** Paeχψ* or ***(D)** Ecoχψ* show a clear increase in the sedimentation coefficients, indicating complex formation. Sedimentation was done at 50000 rpm and 20°C, in high salt buffer (λ=280 nm).
