# Supplementary material for: Characterization of the χψ subcomplex of Pseudomonas aeruginosa DNA polymerase III
Source: BMC Mol Biol. 2011 Sep 28;12:43. doi: 10.1186/1471-2199-12-43 (PMC3197488; doi:10.1186/1471-2199-12-43)
Supplement: Additional file 8 — Figure S8. Sequence alignment of ψ proteins from different members of the Pseudomonadaceae. [file 1471-2199-12-43-S8.PDF]

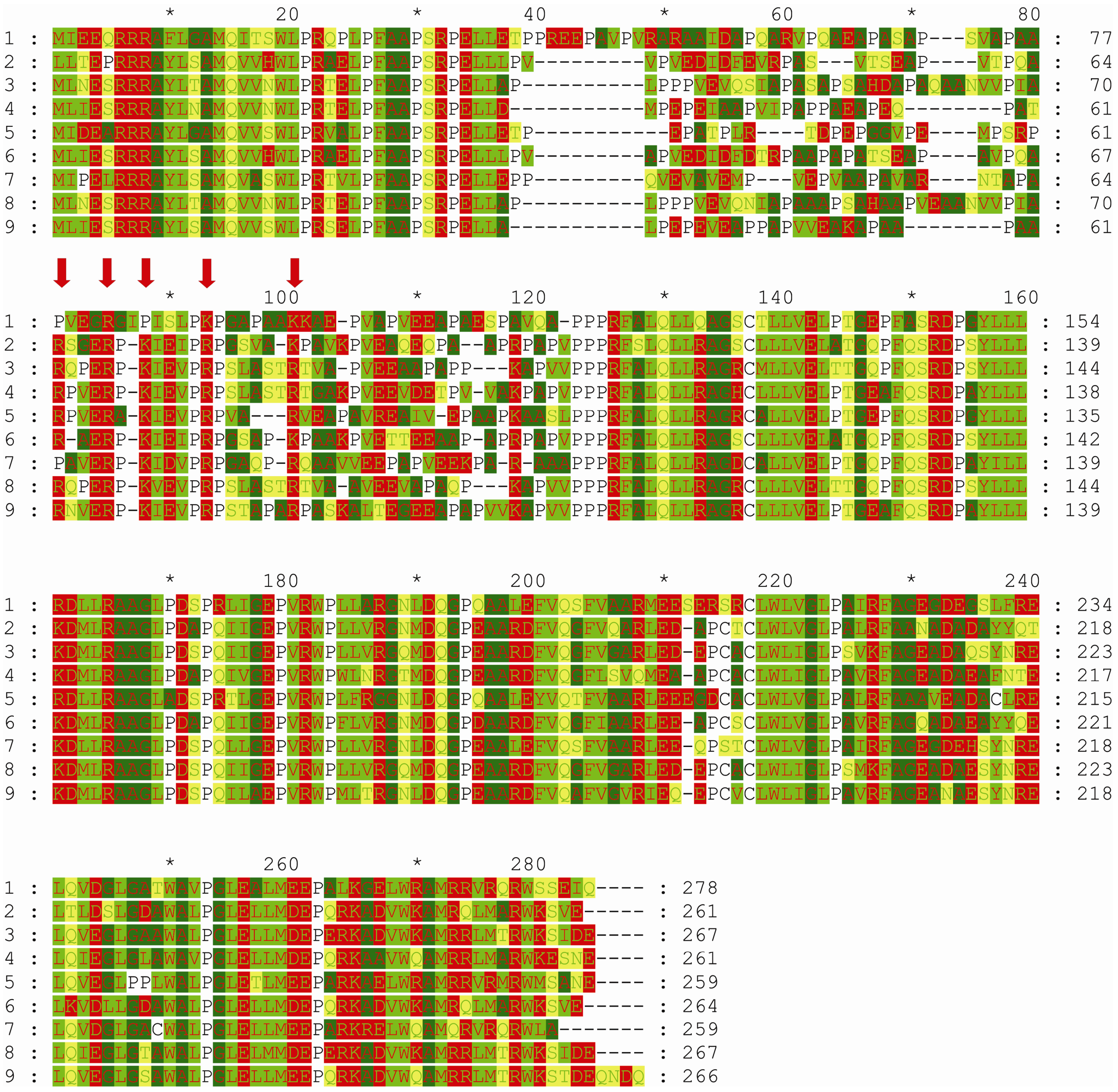

Figure S8. Sequence alignment of ψ proteins from different members of the Pseudomonadaceae. Protein sequences were aligned based on a ClustalW2 result [1] and amino acid residues were colored according to their chemical properties (GeneDoc, http://www.nrbsc.org/). Conserved positively charged residues in the N-terminal region of ψ, possibly implicated in DNA-binding, are marked with red arrows. 1: *P. aeruginosa* PAO1, 2: *P. putida* KT2440, 3: *P. syringae pv. tomato* T1, 4: *P. fluorescens* Pf0-1, 5: *A. vinelandii* DJ / ATCC BAA-1303, 6: *P. entomophila* L48, 7: *P. mendocina ymp*, 8: *P. savastanoi pv. savastanoi* NCPPB 3335, 9: *P. fluorescens* Pf-5 / ATCC BAA-477

1.Goujon M, McWilliam H, Li W, Valentin F, Squizzato S, Paern J, Lopez R: **A new bioinformatics analysis tools framework at EMBL-EBI.** Nucleic Acids Res 2010, **38** (Web Server issue):W695-9.
