# Supplementary material for: Characterization of the χψ subcomplex of Pseudomonas aeruginosa DNA polymerase III
Source: BMC Mol Biol. 2011 Sep 28;12:43. doi: 10.1186/1471-2199-12-43 (PMC3197488; doi:10.1186/1471-2199-12-43)
Supplement: Additional file 9 — Primer sequences used for cloning of the χ and ψ genes of P. aeruginosa, E. coli and P. putida. [file 1471-2199-12-43-S9.PDF]

Primer sequences used for cloning of the χ and ψ genes of *P. aeruginosa, E. coli* and *P. putida*. The respective restriction sites are underlined.

paeχ_for 5′AGGAGGTCTCGCATGACCCGCGTCGATTTCTACGTGATCCCC 3′

paeχ _rev 5′AGGAGGATCCTTAGATACGCGGCAGGCGATGGTC 3′

paeψ_for 5′AGGAAGATCTATCGAAGAACAGCGTCGCCGC 3′

paeψ_rev 5′AGGAGAATTCTTATTGAATCTCGCTCGACCAGC 3′

ecoχ_pET_for 5′ CGGAAGCCCATGGCAAACGCGACGTTCTACCTTCTGGACAAT 3′

ecoχ_pET_rev 5′ GGAAGCTCGAGTCATTTCCAGGTTGCCGTATTCAGGTTGAAA 3′

ecoψ_pET_for 5′ AGGACTCGAGAGGAGATATACCATGACATCCCG 3′

ecoψ_pET_rev 5′ AGGACTCGAGTCAGTCGTTTCGAGGGAAGAAATC 3′

ecoχ_pCDF_for 5′AGGACATATGAAAAACGCGACGTTCTACCTTCTG 3′

ecoχ_pCDF_rev 5′AGGACTCGAGTCATTTCCAGGTTGCCGT 3′

ppuχ_for 5′ AGGAGGTCTCGCATGAGCAAAGTCGACTTCTACATTCTGCCC 3′

ppuχ_rev 5′ AGGAGGATCCTTAAAGTCGCTGTAAGCGGTGGTC 3′

ppuψ_for 5′ ATTAGGATCCCTGACCGAACCCCGTCGCCGCGC 3′

ppuψ_rev 5′ AGGACTCGAGTTATTCAACGCTCTTCCAGCGCG 3′

pae$\psi_{(\Delta 1\text{-}85)}$_for 5′AGGAAGATCTATCAGCCTGCCGAAGCCGGGC 3′

pae$\psi_{(\Delta 1\text{-}85)}$_rev 5′AGGAGAATTCTTATTGAATCTCGCTCGACCAGC 3′
